# Stabilising function of the human diaphragm in response to involuntary augmented breaths induced with or without lower‐limb movements

**DOI:** 10.1113/EP090605

**Published:** 2022-10-21

**Authors:** Camilla R. Illidi, Lee M. Romer

**Affiliations:** ^1^ Division of Sport Health and Exercise Sciences College of Health Medicine and Life Sciences Brunel University London Uxbridge UK

**Keywords:** CO_2_‐rebreathe, cycle ergometry, diaphragm, exercise, hypercapnia, respiratory mechanics, respiratory muscle, ultrasound

## Abstract

**New Findings:**

- **What is the central question of this study?**
  Is the stabilising function of the diaphragm altered differentially in response to involuntary augmented breaths induced with or without lower‐limb movements?
- **What is the main finding and its importance?**
  At equivalent levels of ventilation, the diaphragm generated higher passive pressure but moved significantly less during incremental cycle ergometry compared with progressive hypercapnia. Diaphragm excursion velocity and power output did not differ between the two tasks. These findings imply that the power output of the diaphragm during stabilising tasks involving the lower limbs may be preserved via coordinated changes in contractile shortening.

**Abstract:**

Activity of key respiratory muscles, such as the diaphragm, must balance the demands of ventilation with the maintenance of stable posture. Our aim was to test whether the stabilising function of the diaphragm would be altered differentially in response to involuntary augmented breaths induced with or without lower‐limb movements. Ten healthy volunteers (age 21 (2) years; mean (SD)) performed progressive CO_2_‐rebreathe (5% CO_2_, 95% O_2_) followed 20 min later by incremental cycle exercise (15–30 W/min), both in a semi‐recumbent position. Ventilatory indices, intrathoracic pressures and ultrasonographic measures of diaphragm shortening were assessed before, during and after each task. From rest to iso‐time, inspiratory tidal volume and minute ventilation increased two‐ to threefold. At equivalent levels of tidal volume and minute ventilation, mean inspiratory transdiaphragmatic pressure (${\bar{P}}_{\mathrm{di}}$) was consistently higher during exercise compared with CO_2_‐rebreathe due to larger increases in gastric pressure and the passive component of ${\bar{P}}_{\mathrm{di}}$ (i.e., mechanical output due to static contractions), and yet diaphragm excursion was consistently lower. This lower excursion during exercise was accompanied by a reduction in excursion time with no difference in the active component of ${\bar{P}}_{\mathrm{di}}$. Consequently, the rates of increase in excursion velocity (excursion/time) and power output (active ${\bar{P}}_{\mathrm{di}}$ × velocity) did not differ between the two tasks. In conclusion, the power output of the human diaphragm during dynamic lower‐limb exercise appears to be preserved via coordinated changes in contractile shortening. The findings may have significance in settings where the ventilatory and stabilising functions of the diaphragm must be balanced (e.g., rehabilitation).

## 1 INTRODUCTION

The diaphragm is the principal muscle of inspiration in humans (Grimby et al., 1976). During contraction, the diaphragm descends and flattens thereby expanding the lower ribcage and generating the negative pressure gradient that drives inspiratory flow. In addition to inspiration, the diaphragm muscle is involved in trunk stability during anticipatory postural adjustments (Hodges et al., 1997) and volitional upper‐limb movements (Hodges & Gandevia, 2000a). Specifically, diaphragm descent increases intra‐abdominal pressure, which, in turn, stabilises the spine independently from the muscle's respiratory activity (Hodges & Gandevia, 2000b). Trunk stability is important for maximizing force generation and minimizing joint loads in all dynamic activities, ranging from simple functional tasks to complex athletic movements (Hodges et al., 2004; Oddsson, 1988; see also Kibler et al., 2006). While previous research has provided valuable insight into the mechanisms for coordination of postural and respiratory functions of the diaphragm (Hodges et al., 1997; Hodges & Gandevia, 2000a, 2000b), the data were typically derived from repetitive arm movements (i.e., non‐functional tasks). Whether the findings extend to dynamic lower‐limb exercise is not yet clear.

The reference method for the assessment of diaphragm function is the measurement of transdiaphragmatic pressure (*P*_di_), defined as the difference between pleural and abdominal pressure inferred from catheter‐derived measures of oesophageal (*P*_oe_) and gastric pressure (*P*_ga_), respectively (Laveneziana et al., 2019). A major limitation, however, is that *P*_di_ is merely an index of force output – it is a relatively poor index of contractile function (ATS/ERS, 2002; Laveneziana et al., 2019). The importance of this methodological limitation is emphasised by the finding that during dynamic lower‐limb exercise (cycle ergometry) the diaphragm operates as a ‘flow generator’ (Aliverti et al., 1997); that is, the mechanical power of the diaphragm is mainly a function of shortening velocity rather than force/pressure. Imaging techniques, including fluoroscopy and magnetic resonance imaging (MRI), have been used to provide functional information regarding diaphragm shortening during static postural tasks (Kolar et al., 2012, 2010; Vostatek et al., 2013). Unfortunately, fluoroscopy involves exposure to ionising radiation and MRI is influenced by motion artefacts. Thus, the applicability of these imaging techniques to dynamic exercise is limited.

In recent years, subcostal ultrasonography has emerged as a promising new technique for the quantification of diaphragm shortening. The technique can be used to quantify the mobility (excursion) of the crural diaphragm during tidal or forced inspiration (Laursen et al., 2021), and, if used in combination with established measures, could be used to provide estimates of diaphragm work (force [*P*_di_] × distance [excursion]) and power output (*P*_di_ × velocity [excursion/time]). Traditionally, ultrasonography has been used to evaluate diaphragm dysfunction and weaning outcome in critically ill patients (see Laursen et al., 2021). More recently, the technique has been used to better understand the associations between diaphragm mobility at rest and exertional dyspnoea, exercise limitation and health‐related quality of life in patients with respiratory disease (Crimi et al., 2018; Santana et al., 2019). Diaphragm ultrasonography can be performed repeatedly without adverse effects (see ‘Technical considerations’), and, as such, has the potential to provide new insight into the stabilising function of the diaphragm during dynamic lower‐limb exercise.

The aim of the present study was to test the hypothesis that the stabilising function of the diaphragm would be altered differentially in response to involuntary augmented breaths induced with or without lower‐limb movements in healthy humans. To test this hypothesis, we used subcostal ultrasonography in combination with conventional measures (*P*_di_) to quantify the dynamic contractile function of the human diaphragm in response to progressive hypercapnia versus incremental cycle ergometry.

## 2 METHODS

### 2.1 Ethical approval

The study received ethical approval from the Brunel University London Research Ethics Committee (8404‐A‐Aug/2018‐13769‐1). It conformed to the standards set by the *Declaration of Helsinki*, except for registration in a database (World Medical Association, 2013). All participants gave written informed consent.

### 2.2 Participants

Ten healthy, recreationally active adults (five male and five female) with no history of smoking or cardiorespiratory disease were recruited. Additional inclusion criteria were: age 18−30 years, body mass index 18.5−30.0 kg/m^2^ and pulmonary function values above the lower limit of normal (see below). Participants abstained from strenuous exercise and alcohol for at least 24 h, caffeine for 12 h and food for 3 h before testing.

### 2.3 Experimental overview

Participants visited the laboratory on two occasions. The first visit was for screening, assessment of baseline characteristics and familiarisation with the experimental protocols (including instrumented CO_2_‐rebreathe and exercise tests). The second visit was the experimental trial, which consisted of involuntary augmented breaths induced by progressive hypercapnia (CO_2_‐rebreathe) and incremental cycle ergometry (exercise). The two tasks were performed in a pre‐determined order (CO_2_‐rebreathe, 20 min rest, exercise) to negate any potential influence of exercise‐induced respiratory muscle work or fatigue on ventilatory responses to CO_2_ (Mador & Tobin, 1992). Ventilatory and pulmonary gas‐exchange indices, intrathoracic pressures and ultrasonographic measures of diaphragm shortening were assessed at regular intervals before, during and after each task.

### 2.4 Visit 1

#### 2.4.1 Anthropometric measures

Erect stature and semi‐nude body mass were assessed using an electronic measuring station (Seca 798, Hamburg, Germany). Chest circumference was measured using a retractable tape (Seca 201). Chest depth and chest width were measured using an anthropometer (Harpenden, Holtain Ltd, Crymych, UK). All chest dimensions were measured at relaxation volume (i.e., functional residual capacity, FRC), at the level of the xiphoid process, with the participant in standard anatomical position.

#### 2.4.2 Pulmonary function

Maximum volumes, capacities and flows were assessed using spirometry and body plethysmography (MasterScreen PFT, CareFusion, Hoechberg, Germany) (Graham et al., 2019; M. Miller et al., 2005; Wanger et al., 2005). Maximum inspiratory and expiratory pressure (PI_max_ and PE_max_) were assessed using an electro‐manometer (MicroRPM, CareFusion) and recorded as the highest 1‐s plateau pressure generated during maximal inspiratory and expiratory efforts initiated from residual volume (RV) and total lung capacity (TLC), respectively (Green et al., 2002). Values were expressed in absolute units and as a percentage of predicted (Evans & Whitelaw, 2009; Quanjer et al., 2012; Stocks & Quanjer, 1995).

#### 2.4.3 Diaphragm thickness, thickening and excursion

Thickness, thickening and excursion of the diaphragm were assessed using ultrasonography (Vivid 7 Pro, GE Medical, Horten, Norway). Participants adopted a semi‐recumbent position. Absolute thickness of the right costal hemidiaphragm was measured at FRC and TLC using a high‐frequency, linear transducer (4.0−11.0 MHz, 10L, GE Medical) as per established guidelines (Laursen et al., 2021). Briefly, the transducer was positioned perpendicular to the right anterior or mid‐axillary line at the 8−10th intercostal space and angled medially so that the hemidiaphragm was imaged as a three‐layered structure. The intercostal space with the least lung obscuration at TLC was selected. Ultrasound cine‐loops were recorded in B (brightness)‐mode and analysed offline as still‐images (EchoPac v6.1, GE Medical). Thickness of the right hemidiaphragm was measured, in triplicate, medially between two adjacent ribs between the inner edges of the pleural and peritoneal membranes. Two participants exhibited lung obscuration at TLC; for these individuals, diaphragm thickness was measured as close to TLC as possible. The relative thickening fraction of the right costal hemidiaphragm was calculated as: (thickness at TLC – thickness at FRC)/thickness at FRC (Laursen et al., 2021). Maximum diaphragm excursion, defined as the maximum excursion of the right crural hemidiaphragm from FRC to TLC, was measured using a low‐frequency, curvilinear transducer (2.4−5.0 MHz, 3.5C, GE Medical) as per established guidelines (Laursen et al., 2021). The transducer was positioned subcostally on the right mid‐clavicular line and angled cranially to image the right crural hemidiaphragm from the costophrenic angle to the inferior vena cava. Excursion was measured using angle‐independent (anatomic) M (motion)‐mode (additional details below).

### 2.5 Visit 2

#### 2.5.1 CO_2_‐rebreathe task

The CO_2_‐rebreathe protocol was similar to Read's hyperoxic, hypercapnic rebreathe method (Read, 1967). The participant rested for 10 min in semi‐Fowler's position (30° hip angle with legs straight and arms resting along the torso) while breathing ambient air without a mouthpiece to ensure that the test was initiated from a relaxed, wakeful state (Spengler & Shea, 2001). Next, the participant was fitted with a nose‐clip and breathed for 3 min through a mouthpiece‐valve assembly (2110, Hans Rudolph, Shawnee, KA, USA; 53.5 ml dead space), with resting measurements made over the final 30 s. The participant then exhaled to RV, at which point the rebreathe valve was shut and the participant equilibrated with the now‐closed rebreathe circuit by taking three deep, rapid breaths from a latex‐rubber rebreathe bag (bag volume ≈ vital capacity + 1 litre) filled with a certified gas containing 95% O_2_ and 5% CO_2_ (BOC, Guilford, UK) followed by the instruction to ‘close your eyes, relax and breathe as needed’. When end‐tidal partial pressure for CO_2_ ($P_{\mathrm{ETC}O_{2}}$) reached 55 mmHg, the rebreathe circuit was opened to ambient air and the participant continued to breathe for 3 min (recovery). The laboratory was kept silent before, during and after the rebreathe task to prevent fluctuations in arousal level and the influence of participant–experimenter interaction (Homma & Masaoka, 2008; Spengler & Shea, 2001).

#### 2.5.2 Exercise task

The exercise task was performed on an electromagnetically braked, semi‐recumbent cycle ergometer (Angio imaging no. 967931, Lode, Groningen, The Netherlands) set to hyperbolic mode (i.e., work rate independent of pedal rate). The participant adopted the same position as described for the CO_2_‐rebreathe task. After 10 min of quiet breathing, the participant was fitted with a nose‐clip and breathed through the mouthpiece‐valve assembly for 3 min with their feet attached to pedals via toeclips. As before, resting measurements were made over the final 30 s. Exercise was initiated at 10 W with a cadence of 55−60 rpm for 3 min (warm‐up), after which the work rate was increased as a ramp by 18–30 W/min. The pedal cadence was chosen to minimise the lateral motion of torso and hips during ultrasound imaging. The ramp rate was determined for each participant by calculating the predicted peak oxygen uptake (${\dot{V}}_{O_{2}\mathrm{peak}}$; l/min) for a work efficiency (∆${\dot{V}}_{O_{2}}$/∆WR) of 10 ml/min/W over a test duration of 8 min (Wasserman et al., 2012, p.158). This duration was chosen on the basis that it was near the expected duration for CO_2_‐rebreathe, yet still at the lower end of the range expected to elicit maximum physiological responses (Buchfuhrer et al., 1983). The exercise was terminated when pedal cadence dropped below 50 rpm for more than 5 s despite verbal encouragement. The participant continued to breathe on the mouthpiece for a further 3 min for the collection of recovery data.

#### 2.5.3 Perceptual ratings

At the end of CO_2_‐rebreathe and exercise, participants were asked to rate their dyspnoea intensity (‘How strong/intense is your breathing discomfort?’) using Borg's modified category ratio scale (CR‐10; Borg, 1998). At the end of exercise, participants were also asked to rate their intensity of leg discomfort using the CR‐10 scale. The scale was anchored at 0 (‘no breathing [leg] discomfort’) and 10 (‘the most severe breathing [leg] discomfort ever experienced or imagined’). Finally, participants were asked to verbalise their main reasons for exercise cessation (breathing, legs, combination of both, other reasons).

#### 2.5.4 Ventilatory and pulmonary gas‐exchange indices

Ventilatory and pulmonary gas‐exchange indices were assessed breath‐by‐breath using an online system that comprised a turbine flow meter and O_2_ and CO_2_ gas analysers (Oxycon Pro, Jaeger, Viasys Healthcare, Hoechberg, Germany). The flow meter and gas analysers were calibrated immediately before each task according to the manufacturer's recommendations. Raw signals from the online system were input into a data acquisition system (Micro1401‐2 and Spike2, CED, Cambridge, UK) using an external device (DAQ‐30A16, Eagle Technology, Cape Town, South Africa) for the offline determination of inspiratory minute ventilation (${\dot{V}}_{I}$, BTPS), inspiratory tidal volume (*V*_TI_, BTPS), respiratory frequency (ƒ_R_), inspiratory time (*T*_I_), and inspiratory duty cycle (*T*_I_/*T*_TOT_) (see below). Metabolic and gas‐exchange indices (${\dot{V}}_{O_{2}}$, $P_{\mathrm{ETC}O_{2}}$, etc.) were taken directly from the online system.

#### 2.5.5 Intrathoracic pressures

Oesophageal and gastric pressure (*P*_oe_ and *P*_ga_) were measured using two balloon‐tipped catheters (CooperSurgical, Berlin, Germany). Placement of the catheters was preceded by the administration of 2% lidocaine gel to the nasal mucosa. Each catheter was passed via the nares and swallowed. Once in the stomach, each catheter was connected to a low‐range differential pressure transducer (DP45‐3, Validyne Engineering, Northridge, CA, USA) that was calibrated across the physiological range using a digital manometer (C9553, JMW, Harlow, UK). The balloons were filled with 1 ml (*P*_oe_) or 2 ml (*P*_ga_) of air using a 10 ml glass syringe. The oesophageal catheter was withdrawn from the stomach until a negative deflection in *P*_oe_ was observed upon inspiration. The catheter was withdrawn a further 10 cm so that the distal end was situated in the lower one‐third of the oesophagus. A dynamic occlusion test was performed to confirm the oesophageal catheter was in the correct position (Baydur et al., 1982), after which, both catheters were taped in position at the nose. The volume of air in each catheter was checked at regular intervals to ensure no leaks had occurred. Analogue pressure signals were amplified (CD280, Validyne), digitised (Micro1401‐2, CED), recorded online at 200 Hz (Spike2, CED), then down‐sampled to correspond with the ventilatory signals (100 Hz). Instantaneous transdiaphragmatic pressure was calculated online (*P*_di_ = *P*_ga_ − *P*_oe_).

#### 2.5.6 Diaphragm shortening

Images of crural diaphragm shortening were obtained using a low‐frequency, convex‐array ultrasound transducer (2.4−5.0 MHz, 3.5C, GE Medical) which facilitated a wide field of view with adequate frame‐rate of capture (40−60 frames/s). The transducer was positioned subcostally on the right mid‐clavicular line, as per established procedures (Laursen et al., 2021). Here, the right hemidiaphragm dome was imaged at the interface of the visceral pleura and air‐filled lung. Beam penetration depth was adjusted so that the hyperechoic diaphragm at RV was always within the field of view (∼200−250 mm). The inferior vena cava was used as an anatomical landmark to ensure the region of interest and the insonation angle were kept constant. To optimise lateral resolution, one focal point was set at the diaphragm position at relaxation volume. The site of transducer placement was clearly marked with indelible ink to ensure replication during subsequent scans.

To minimise movement of the transducer, a step stool was used to rest the sonographer's arm and wrist on their knee during imaging. In addition, two ECG electrodes (3M, Bracknell, UK) were placed on the right and left mid‐axillary lines so that the ultrasound system could monitor changes in ribcage volume via changes in distance between the two electrodes. The waveform signal of ribcage volume shown online during scanning and offline during analysis was used to distinguish between inspiration and expiration (see below). To ensure consistency, the same experimenter (C.I.) performed all assessments.

**FIGURE 1:**
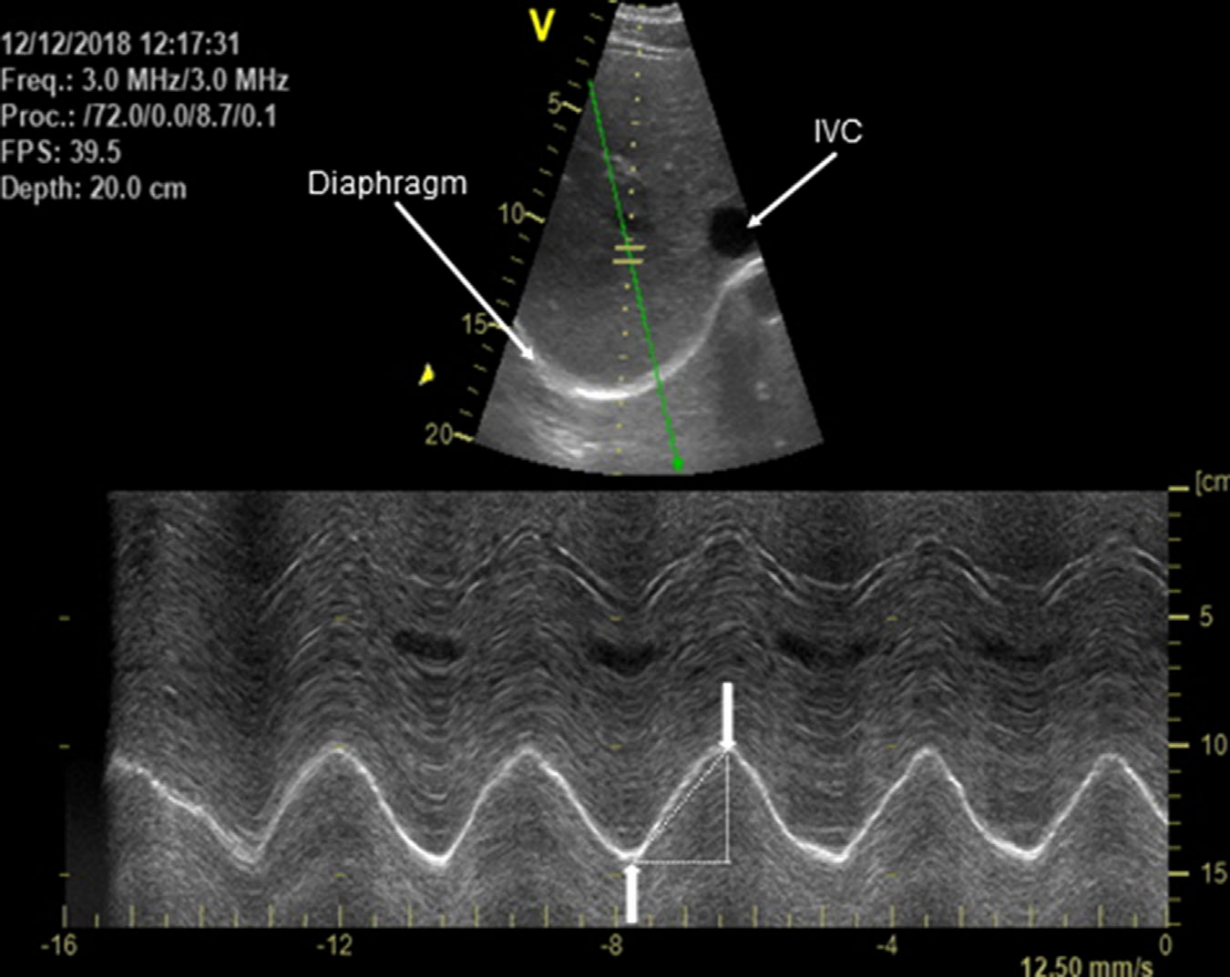
Representative ultrasound images of the right hemidiaphragm during the final 15 s of CO_2_‐rebreathe ($P_{\mathrm{ETC}O_{2}}$, 55 mmHg; ${\dot{V}}_{I}$ 32 l/min). B‐mode image (top) was initially used to obtain the best diaphragmatic delineation, with the inferior vena cava (IVC) used as an anatomical landmark to ensure consistency in the positioning of the transducer. In anatomic M‐mode (bottom), the diaphragm excursion (cm), excursion velocity (slope, cm/s) and excursion time (s) were measured for the estimation of diaphragm work and power output (see text for details). Vertical arrows indicate the beginning and end of a diaphragm contraction

Ultrasound cine‐loops were recorded in B‐mode, then analysed offline using angle‐independent (anatomic) M‐mode ultrasound (EchoPac v6.1, GE Medical). The dynamic cursor was orientated along the true axis of diaphragm movement (Orde et al., 2016). Acceptable cine‐loops were characterised by clear hyperechoic lines that distinctively marked the start and the end of an inspiratory cycle. Inspiratory diaphragm excursion and excursion time were measured with a digital calliper as the absolute excursion amplitude and the time from onset of excursion to peak excursion, respectively. The measurements were always made from one side of the anatomic M‐mode trace. Mean diaphragm excursion velocity during inspiration was calculated as the quotient of diaphragm excursion and excursion time. Diaphragm work and power output during inspiration were estimated as force (active mean *P*_di_) × distance (excursion) and force (active mean *P*_di_) × velocity (excursion/time), respectively (see also below). Representative recordings from a CO_2_‐rebreathe trial are shown in Figure 1.

#### 2.5.7 Data processing and time‐matching

The start and finish of each breath were marked at points of zero flow. Anomalous breaths (e.g., swallows, coughs, sighs or breaths not crossing zero flow) were manually excluded. Inspired and expired volumes were derived via numerical integration of flow. Tidal pressures were expressed as mean inspiratory pressures (${\bar{P}}_{\mathrm{di}}$, ${\bar{P}}_{\mathrm{ga}}$ and ${\bar{P}}_{\mathrm{oe}}$). For instance, ${\bar{P}}_{\mathrm{di}}$ was calculated as $\Sigma_{0}^{n}$(*P*_di_/*n*), where *P*_di_ (0) was the absolute *P*_di_ at the onset of inspiration and *n* was the number of data points during inspiration (Barnard & Levine, 1986). The active component of ${\bar{P}}_{\mathrm{di}}$ (${\bar{P}}_{\mathrm{di},a}$) was calculated by subtracting the lowest pressure during any given respiratory cycle from the instantaneous pressure. The passive component (${\bar{P}}_{\mathrm{di},p}$) was calculated by subtracting ${\bar{P}}_{\mathrm{di},a}$ from ${\bar{P}}_{\mathrm{di}}$. Accordingly, ${\bar{P}}_{\mathrm{di},a}$ was the pressure required to accomplish inspiration (i.e., dynamic contractions), whereas ${\bar{P}}_{\mathrm{di},p}$ was the pressure required to brace and stabilise the trunk (i.e., static contractions). The ratio ${\bar{P}}_{\mathrm{di}}$/${\bar{P}}_{\mathrm{oe}}$ (active pressures) was used to estimate the pressure contribution of ribcage muscles relative to that of the diaphragm during inspiration (Yan & Kayser, 1997). End‐expiratory and end‐inspiratory oesophageal pressure (EE*P*_oe_ and EI*P*_oe_) were used as indexes of end‐expiratory and end‐inspiratory lung volume (EELV and EILV), respectively.

To ensure the respiratory cycles within each ultrasound cine‐loop were correctly matched with the breath‐by‐breath ventilatory and pressure data, particular attention was paid to the timing of ultrasound acquisition. Specifically, 15 s ultrasound cine‐loops were recorded twice at rest, then every 30 s during hyperpnoea and recovery. All breaths recorded within a cine‐loop were identified in the data‐acquisition system and averaged over 15 s. Since all participants achieved a test duration of at least 1.75 min, data up to and including this time point (iso‐time) were used for analysis. The final 15 s of hyperpnoea was also recorded, and defined as the peak response.

### 2.6 Statistics

Statistical analysis was performed using dedicated software (SPSS v26.0, IBM Corp., Armonk, NY, USA; GraphPad Prism v9.3, GraphPad Software, San Diego, CA, USA). All data were tested for normality and equality of variance using the Shapiro–Wilk test and Levene's test, respectively. The effect of task (CO_2_‐rebreathe, exercise) and sex (male, female) on selected variables was analysed using Student's paired *t*‐test. Feasibility of ultrasonography for the assessment of diaphragm shortening was quantified as the percentage of technically acceptable cine‐loops (i.e., cine‐loops with acceptable image quality and without excessive motion artefact). Ventilatory and pulmonary gas‐exchange indices, intrathoracic pressures and ultrasonographic measures were compared using two‐way repeated measures ANOVA (time [rest, 0.75 min, 1.25 min, 1.75 min iso‐time, peak] × task [CO_2_‐rebreathe, exercise]) with Holm–Šidák correction for multiple pairwise comparisons. Mauchly's test was used to determine sphericity, and Greenhouse–Geisser correction was applied when sphericity was violated. Effect sizes were reported as partial eta squared ($\eta_{p}^{2}$), and interpreted as small ($\eta_{p}^{2}$ = 0.01), medium ($\eta_{p}^{2}$ = 0.06) or large ($\eta_{p}^{2}$ = 0.14) (Lakens, 2013). The rate of change (i.e., slope) in diaphragm shortening (excursion and excursion velocity) versus ventilatory parameters (*V*_TI_ and *V*_TI_/*T*_I_) was determined for group mean data using linear regression. Slopes for the two tasks were compared using the calculations provided by Zar (2014). Descriptive data are reported as mean (SD). Statistical significance was set at *P* ≤ 0.05.

## 3 RESULTS

### 3.1 Participant characteristics

Participant characteristics are shown in Table 1. As expected, males were taller and heavier than females and exhibited larger chest dimensions. Static and dynamic lung volumes and maximum expiratory pressure were higher in males compared with females. However, all participants exhibited pulmonary function within normal limits. Diaphragm thickness was similar for both sexes, whereas maximum diaphragm excursion was greater in males.

**TABLE 1:** Participant characteristics

	Total (*n* = 10)	Males (*n* = 5)	Females (*n* = 5)
Anthropometric measures			
Age (years)	21 (2)	22 (2)	21 (2)
Stature (cm)	167 (11)	175 (8)	160 (9)*
Body mass (kg)	66.3 (11.7)	77.5 (4.3)	57.4 (6.3)*
Body mass index (kg/m^2^)	23.5 (2.6)	25.2 (3.1)	22.3 (0.6)
Chest circumference (cm)	82.9 (9.8)	91.8 (5.3)	75.8 (5.2)*
Chest depth (cm)	19.6 (2.3)	21.2 (2.6)	18.3 (1.0)*
Chest width (cm)	27.5 (2.7)	29.5 (0.6)	25.6 (2.6)*
Pulmonary function			
TLC (l)	5.97 (1.27) [100 (9)]	7.11 (0.17) [102 (17)]	5.02 (0.96)* [96 (3)]
RV (l)	1.56 (0.36) [118 (27)]	1.60 (0.36) [115 (33)]	1.41 (0.40) [120 (25)]
FRC_pleth_ (l)	3.16 (0.85) [104 (18)]	3.72 (0.88) [118 (24)]	2.51 (0.54)* [91 (13)]
FVC (l)	4.71 (1.09) [108 (6)]	5.69 (0.23) [107 (7)]	3.92 (0.80)* [106 (9)]
FEV_1_ (l)	3.98 (0.84 [104 (8)]	4.69 (0.25) [104 (8)]	3.40 (0.68)* [104 (10)]
FEV_1_/FVC	0.84 (0.03) [97 (3)]	0.82 (0.02) [96 (3)]	0.86 (0.02)* [98 (3)]
MVV_12_ (l/min)	147 (23) [94 (13)]	163 (10) [89 (10)]	134 (23)* [100 (13)]
PI_max_ (cmH_2_O)	−120 (25) [111 (21)]	−132 (24) [115 (2)]	−111 (25) [109 (20)]
PE_max_ (cmH_2_O)	156 (38) [121 (19)]	187 (34) [114 (23)]	134 (23)* [129 (10)]
Diaphragm characteristics			
Thickness at FRC (mm)	1.3 (0.2)	1.4 (0.1)	1.2 (0.1)*
Thickness at TLC (mm)	3.9 (0.9)	4.4 (0.4)	3.5 (0.4)*
Thickening fraction (%)	287 (90)	337 (35)	245 (42)*
Maximum excursion (cm)	6.35 (0.58)	6.53 (0.55)	6.17 (0.61)

Data are means (SD). Values in square brackets are % predicted.

* *P* ≤ 0.05 versus males. FEV_1_, forced expiratory volume in 1 s; FRC_pleth_, plethysmography‐derived functional residual capacity; FVC, forced vital capacity; MVV_12_, maximum voluntary ventilation in 12 s; PE_max_, maximum expiratory mouth pressure; PI_max_, maximum inspiratory mouth pressure; RV, residual volume; TLC, total lung capacity.

### 3.2 Responses to CO_2_‐rebreathe and exercise

All participants terminated CO_2_‐rebreathe at the standardised cut‐off of 55 mmHg without untoward events. Test duration was shorter for CO_2_‐rebreathe compared with exercise [3.1 (1.1) vs. 8.1 (1.8) min, respectively; *P* < 0.001]. Participants achieved the following peak values for exercise: work rate 172 (50) W, ${\dot{V}}_{O_{2}}$ 2.81 (0.88) l/min [42.1 (13.2) ml/kg/min, 100 (20)% of estimated ${\dot{V}}_{O_{2}\max}$] and RER 1.09 (0.09). Peak ${\dot{V}}_{I}$ was higher for exercise compared with CO_2_‐rebreathe [70 (23) vs. 30 (12) l/min, *P* < 0.001], with values corresponding to 47 (14)% and 20 (8)% of maximum voluntary ventilation. Intensity of limb discomfort at exercise cessation was 7.8 (1.9), with 70% of participants indicating legs as their main reason for stopping and the remaining 30% indicating a combination of breathing and legs. Peak dyspnoea intensity was higher for exercise compared with CO_2_‐rebreathe [5.5 (2.8) vs. 4.0 (2.0), *P* = 0.016].

#### 3.2.1 Ventilatory responses

**FIGURE 2:**
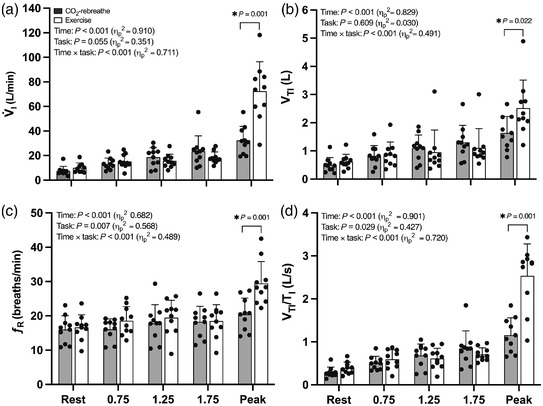
Ventilatory and breathing pattern responses to CO_2_‐rebreathe and exercise. Data are means (SD) for 10 participants. See text for abbreviations

Ventilatory and breathing pattern responses to CO_2_‐rebreathe and exercise are shown in Figure 2 (see also Table 2). On average, inspiratory minute ventilation (${\dot{V}}_{I}$) increased by a factor of 2.4 between rest and 1.75 min (iso‐time). The values at each time point were not significantly different between the two tasks (Figure 2a). The initial increases in ${\dot{V}}_{I}$ were achieved via progressive increases in tidal volume (Figure 2b) and respiratory frequency (Figure 2c). Tidal volume was slightly lower at the later time points for exercise versus CO‐rebreathe, but there were no significant differences between the two tasks. The increases in tidal volume during CO_2_‐rebreathe and exercise were accompanied by similar increases in inspiratory time (*T*_I_). As a result, mean inspiratory flow (*V*_T_/*T*_I_) also did not differ between the two tasks (Figure 2d). At peak, the ventilatory responses were greater for exercise versus CO_2_‐rebreathe. During recovery, the ventilatory responses returned towards resting baseline but remained consistently elevated for exercise versus CO_2_‐rebreathe (data not shown).

**TABLE 2:** Ventilatory and breathing pattern responses to CO_2_‐rebreathe and exercise

	Rest		0.75 min		1.25 min		1.75 min (iso‐time)		Peak		*P*		
	CO_2_	Exercise	CO_2_	Exercise	CO_2_	Exercise	CO_2_	Exercise	CO_2_	Exercise	Time	Task	Time × Task
${\dot{V}}_{I}$ (l/min)	7.8 (3.9)	9.4 (2.6)	12.7 (4.9)	15.3 (5.7)	17.9 (8.1)	15.6 (5.5)	22.3 (13.2)	18.2 (4.8)	30.2 (11.6)	70.1 (22.8)*	**0.000**	0.055	**0.000**
*V*_TI_ (l)	0.52 (0.28)	0.57 (0.16)	0.83 (0.37)	0.87 (0.44)	1.14 (0.48)	0.95 (0.78)	1.34 (0.61)	1.10 (0.69)	1.63 (0.58)	2.44 (0.93)*	**0.000**	0.609	**0.000**
*f*_R_ (breaths/min)	15.8 (4.0)	16.9 (3.8)	17.3 (8.1)	18.6 (4.1)	16.8 (6.4)	19.5 (5.0)	17.1 (5.7)	18.5 (4.8)	19.1 (4.8)	29.2 (6.5)*	**0.000**	**0.007**	**0.000**
*T*_I_ ( s)	1.71 (0.56)	1.61 (0.44)	1.72 (0.59)	1.53 (0.47)	1.89 (0.87)	1.55 (0.68)*	1.83 (0.85)	1.53 (0.57)*	1.53 (0.38)	1.03 (0.34)*	**0.028**	**0.013**	**0.021**
*T*_TOT_ (s)	4.02 (1.03)	3.55 (1.29)	3.98 (1.36)	3.40 (0.72)	4.14 (1.83)	3.36 (1.29)	4.00 (1.79)	3.50 (1.20)	3.32 (0.84)	2.13 (0.41)	**0.004**	**0.017**	0.140
*T*_I_/*T*_TOT_	0.42 (0.05)	0.43 (0.03)	0.43 (0.04)	0.45 (0.05)	0.45 (0.04)	0.46 (0.08)	0.45 (0.03)	0.44 (0.05)	0.46 (0.04)	0.48 (0.06)	0.053	0.565	0.681
*V*_TI_/*T*_I_ (l/s)	0.29 (0.12)	0.39 (0.14)	0.48 (0.17)	0.59 (0.22)	0.65 (0.29)	0.61 (0.23)	0.80 (0.43)	0.70 (0.15)	1.10 (0.42)	2.48 (0.78)*	**0.000**	**0.029**	**0.000**

Data are means (SD) for 10 participants.

* *P* ≤ 0.05 versus exercise at given time point as per *post hoc* tests. Values in bold indicate statistical significance. *f*_R_, respiratory frequency; *T*_I_, inspiratory time; *T*_I_/*T*_TOT_, inspiratory duty cycle; *T*_TOT_, total breath time; ${\dot{V}}_{I}$, inspiratory minute ventilation; *V*_TI_, inspiratory tidal volume; *V*_TI_/*T*_I_, mean inspiratory flow.

#### 3.2.2 Respiratory pressure responses

**FIGURE 3:**
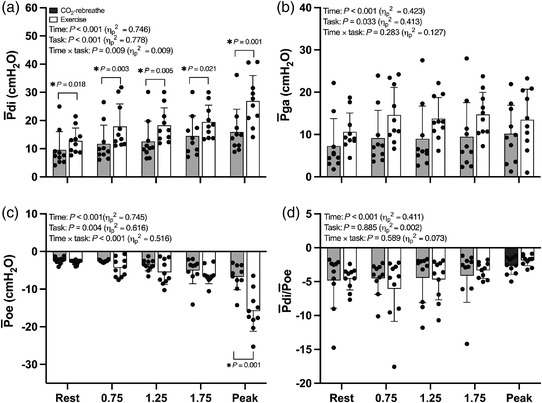
Intrathoracic‐pressure responses to CO_2_‐rebreathe and exercise. Data are means (SD) for 10 participants. See text for abbreviations

**FIGURE 4:**
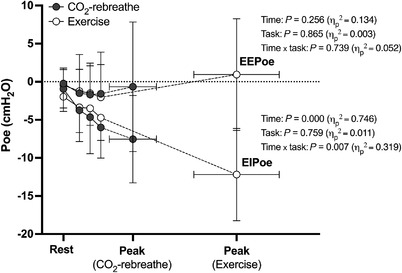
End‐expiratory oesophageal pressure (EE*P*_oe_) and end‐inspiratory oesophageal pressure (EI*P*_oe_) in response to CO_2_‐rebreathe and exercise. The three time points between rest and peak are 0.75 min, 1.25 min and 1.75 min (iso‐time), respectively. Data are means (SD) for 10 participants. ^*^ *P* ≤ 0.05 at given time point as per *post hoc* tests. See text for abbreviations

**FIGURE 5:**
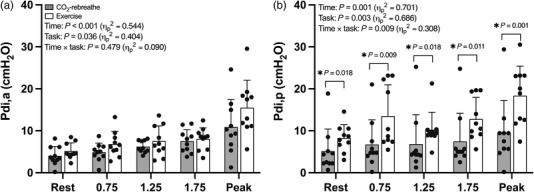
Active and passive components of transdiaphragmatic pressure (${\bar{P}}_{\mathrm{di},a}$ and ${\bar{P}}_{\mathrm{di},p}$) in response to CO_2_‐rebreathe and exercise. Data are means (SD) for 10 participants. See text for abbreviations

Intrathoracic‐pressure responses to CO_2_‐rebreathe and exercise are shown in Figures 3, 4, 5 (see also Table 3). Despite similar ventilatory responses (see above), ${\bar{P}}_{\mathrm{di}}$ was consistently higher during submaximal exercise compared with CO_2_‐rebreathe (∼44%; Figure 3a). These differences in ${\bar{P}}_{\mathrm{di}}$ were largely due to greater increases in ${\bar{P}}_{\mathrm{ga}}$ versus ${\bar{P}}_{\mathrm{oe}}$ (Figure 3b,c). As expected, ${\bar{P}}_{\mathrm{di}}$ at peak was higher for exercise due to the greater ventilatory demand. The ${\bar{P}}_{\mathrm{di}}$/${\bar{P}}_{\mathrm{oe}}$ ratio was stable over time and similar for both tasks (Figure 3d), thereby indicating no differences in the inspiratory pressure contribution of ribcage muscles relative to that of the diaphragm. EE*P*_oe_ did not differ from rest in either task (Figure 4), thereby indicating no changes in end‐expiratory lung volume due to expiratory muscle recruitment or dynamic lung hyperinflation. In contrast, EI*P*_oe_ (a surrogate for end‐inspiratory lung volume) decreased at a faster rate and tended to be less negative (not significant) throughout exercise versus CO_2_‐rebreathe (Figure 4). The greater increases in ${\bar{P}}_{\mathrm{di}}$ during the initial stages of exercise were the result of greater increases in the passive component of ${\bar{P}}_{\mathrm{di}}$, with no differences in the active component (Figure 5a,b). The active component of ${\bar{P}}_{\mathrm{di}}$ during exercise remained elevated into recovery due to the elevated ${\bar{P}}_{\mathrm{di}}$ at peak. In contrast, the passive component of ${\bar{P}}_{\mathrm{di}}$ returned to resting baseline within the first 30 s of exercise cessation.

**TABLE 3:** Intrathoracic‐pressure responses to CO_2_‐rebreathe and exercise

	Rest		0.75 min		1.25 min		1.75 min (iso‐time)		Peak		*P*		
	CO_2_	Exercise	CO_2_	Exercise	CO_2_	Exercise	CO_2_	Exercise	CO_2_	Exercise	Time	Task	Time × Task
${\bar{P}}_{\mathrm{di}}$ (cmH_2_O)	9.6 (6.4)	12.8 (4.6)*	11.7 (6.6)	17.9 (8.0)*	12.6 (7.3)	18.3 (6.2)*	14.5 (7.2)	19.5 (5.9)*	16.0 (8.1)	26.9 (8.6)*	**0.000**	**0.000**	**0.009**
${\bar{P}}_{\mathrm{ga}}$ (cmH_2_O)	9.0 (7.0)	10.0 (4.6)	9.3 (6.5)	14.4 (6.6)	9.8 (7.5)	12.7 (4.8)	10.0 (7.7)	13.6 (5.5)	10.3 (9.3)	14.6 (5.9)	**0.000**	**0.033**	0.283
${\bar{P}}_{\mathrm{oe}}$ (cmH_2_O)	−2.3 (0.9)	−2.9 (0.6)	−2.6 (0.3)	−3.5 (3.6)	−3.6 (1.5)	−5.5 (3.2)	−5.0 (3.5)	−6.4 (2.2)	−6.0 (4.7)	−15.8 (5.4)*	**0.000**	**0.004**	**0.000**
${\bar{P}}_{\mathrm{di}}$/${\bar{P}}_{\mathrm{oe}}$	−4.8 (4.1)	−4.6 (1.7)	−4.5 (2.4)	−6.1 (4.8)	−4.4 (3.7)	−4.7 (3.0)	−4.1 (3.9)	−3.3 (1.2)	−1.9 (2.3)	−1.9 (0.8)	**0.001**	0.885	0.589
EE*P*_oe_ (cmH_2_O)	−0.2 (1.8)	−0.3 (2.3)	−1.6 (3.1)	−1.0 (4.8)	−1.7 (4.1)	−1.5 (3.6)	−1.6 (5.5)	−1.9 (4.3)	−0.7 (8.5)	−0.6 (7.4)	0.256	0.865	0.739
EI*P*_oe_ (cmH_2_O)	−0.9 (2.5)	−2.0 (2.3)	−3.8 (4.1)	−3.4 (3.3)	−4.7 (4.8)	−3.5 (2.2)	−6.0 (4.0)	−4.7 (3.0)	−7.5 (5.7)	−12.2 (6.1)	**0.000**	0.759	**0.007**
${\bar{P}}_{\mathrm{di},a}$ (cmH_2_O)	4.9 (2.0)	5.6 (2.2)	5.9 (2.2)	8.3 (4.4)*	6.6 (2.0)	8.1 (4.8)	7.8 (3.1)	8.7 (4.1)	10.9 (6.5)	17.2 (6.1)*	**0.000**	**0.036**	0.479
${\bar{P}}_{\mathrm{di},p}$ (cmH_2_O)	5.3 (5.7)	8.3 (3.4)*	6.9 (6.2)	14.1 (7.6)*	6.8 (7.7)	10.2 (4.4)*	6.8 (8.0)	13.2 (5.3)*	9.4 (8.4)	19.0 (7.8)*	**0.000**	**0.003**	**0.009**

Data are means (SD) for 10 participants.

* *P* ≤ 0.05 versus exercise at given time point as per *post hoc* tests. Values in bold indicate statistical significance. EE*P*_oe_, end‐expiratory *P*_oe_; EI*P*_oe_, end‐inspiratory oesophageal pressure; ${\bar{P}}_{\mathrm{di}}$, mean inspiratory transdiaphragmatic pressure; ${\bar{P}}_{\mathrm{di}}$/${\bar{P}}_{\mathrm{oe}}$, pressure contribution of ribcage muscles relative to that of the diaphragm during inspiration; ${\bar{P}}_{\mathrm{di},a}$, active component of ${\bar{P}}_{\mathrm{di}}$; ${\bar{P}}_{\mathrm{di},p}$, passive component of ${\bar{P}}_{\mathrm{di}}$; ${\bar{P}}_{\mathrm{ga}}$, mean inspiratory gastric pressure; ${\bar{P}}_{\mathrm{oe}}$, mean inspiratory oesophageal pressure.

#### 3.2.3 Diaphragm shortening

**FIGURE 6:**
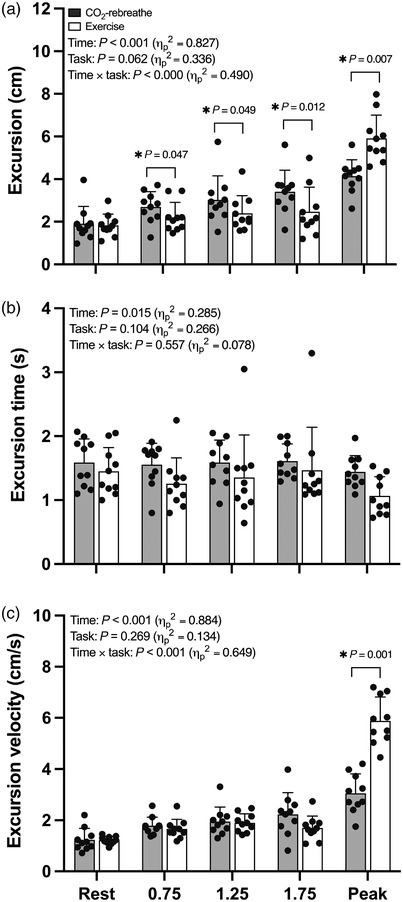
Ultrasonographic measures of diaphragm shortening in response to CO_2_‐rebreathe and exercise. Data are means (SD) for 10 participants. See text for abbreviations

**FIGURE 7:**
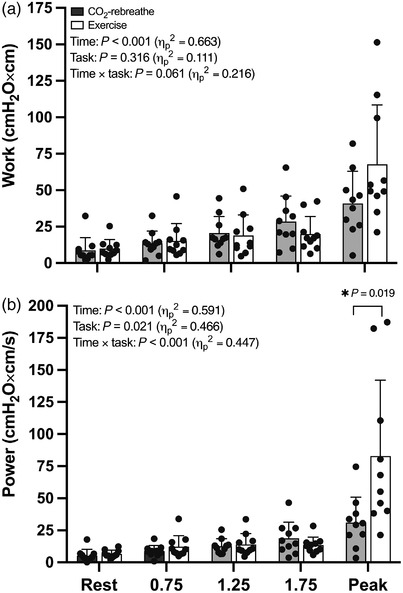
Ultrasound‐derived parameters of diaphragm work and power output in response to CO_2_‐rebreathe and exercise. Data are means (SD) for 10 participants

Ultrasound‐derived measures of diaphragm shortening are shown in Figures 6 and 7 (see also Table 4). A total of 55 and 110 ultrasound cine‐loops were obtained during CO_2_‐rebreathe and exercise, respectively. Of these, 55 (100%) and 106 (96%) were considered technically acceptable. No between‐task differences were noted at rest.

**TABLE 4:** Ultrasonographic measures of diaphragm shortening in response to CO_2_‐rebreathe and exercise

	Rest		0.75 min		1.25 min		1.75 min (iso‐time)		Peak		*P*		
	CO_2_	Exercise	CO_2_	Exercise	CO_2_	Exercise	CO_2_	Exercise	CO_2_	Exercise	Time	Task	Time × Task
Excursion (cm)	1.89 (0.81)	1.79 (0.53)	2.70 (0.72)	2.09 (0.74)*	3.15 (1.19)	2.33 (0.84)*	3.56 (1.13)	2.46 (1.15)*	4.43 (1.29)	5.04 (1.17)*	**0.000**	0.062	**0.000**
Excursion time (s)	1.58 (0.37)	1.44 (0.37)	1.55 (0.33)	1.25 (0.40)	1.58 (0.35)	1.35 (0.66)	1.60 (0.26)	1.46 (0.67)	1.44 (0.37)	1.06 (0.30)	**0.015**	0.104	0.557
Excursion velocity (cm/s)	1.20 (0.45)	1.24 (0.18)	1.75 (0.35)	1.69 (0.38)	1.98 (0.56)	1.82 (0.39)	2.30 (0.87)	1.69 (0.47)	3.11 (0.80)	4.93 (1.16)*	**0.000**	0.269	**0.000**
Work (cmH_2_O × cm)	8.8 (8.7)	9.9 (6.3)	13.6 (8.4)	15.2 (11.9)	20.1 (11.4)	18.9 (14.1)	28.6 (17.4)	19.9 (12.0)	40.9 (21.9)	65.3 (44.4)	**0.000**	0.316	0.061
Power (cmH_2_O × cm/s)	5.3 (4.9)	6.6 (3.1)	8.7 (4.5)	12.2 (8.6)	12.7 (5.8)	13.9 (8.6)	18.7 (12.6)	13.5 (6.2)	31.0 (19.8)	81.0 (62.3)	**0.000**	**0.021**	**0.000**

Data are means (SD) for 10 participants.

* *P* ≤ 0.05 versus exercise at given time point as per *post hoc* tests. Values in bold indicate statistical significance.

**FIGURE 8:**
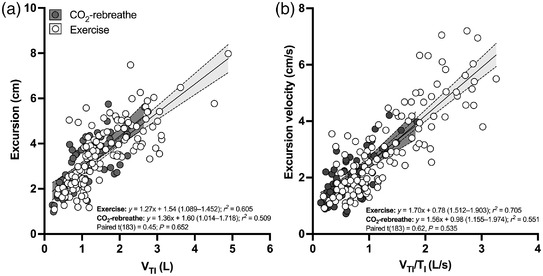
Relationships between ventilatory and ultrasonographic measures of diaphragm shortening in response to CO_2_‐rebreathe and exercise. Data are for 10 participants. Also shown are the linear regression equation (95% confidence interval for slope) and *r* ^2^ value for each task as well as the *t*‐score (degrees of freedom) and *P*‐value for the comparison of slopes. There were no differences in slopes between the two tasks

During CO_2_‐rebreathe, diaphragm excursion increased from 1.89 (0.81) to 4.43 (1.29) cm (30–70% of maximum excursion); that is, by a factor of 2.3 between rest and peak. During exercise, diaphragm excursion increased from 1.79 (0.53) to 5.04 (1.17) cm (28–79% of maximum excursion); that is, by a factor of 2.8. *Post hoc* tests showed that diaphragm excursion was blunted at the first time point during exercise (*P* = 0.047) through iso‐time (*P* = 0.012) (Figure 6a), indicating that the diaphragm moved significantly less during exercise compared with CO_2_‐rebreathe for equivalent levels of tidal volume and minute ventilation. The blunted excursion during submaximal exercise was accompanied by a slightly shorter excursion time (Figure 6b). As a result, excursion velocity (excursion/time) did not differ between the two tasks (Figure 6c). Because the active component of ${\bar{P}}_{\mathrm{di}}$ was slightly higher during submaximal exercise (see ‘Respiratory pressure responses’), diaphragm work (${\bar{P}}_{\mathrm{di},a}$ × excursion) and diaphragm power (${\bar{P}}_{\mathrm{di},a}$ × excursion velocity) also did not differ between the two tasks (Figure 7). Due to the higher ventilatory demand at peak exercise (relative to CO_2_‐rebreathe), the measures of excursion, velocity and power were also higher at peak (Figures 6 and 7) and throughout recovery. Positive linear relationships were noted for *V*_TI_ versus diaphragm excursion and *V*_TI_/*T*_I_ versus excursion velocity (Figure 8); the mean rates of rise (slopes) did not differ between the two tasks, indicating that the blunting of diaphragm excursion during exercise was ‘real’ and not merely artefactual. Sex‐disaggregated data are shown in Table 5. In general, the absolute values were larger in males but the relative changes were qualitatively similar for both sexes.

**TABLE 5:** Ultrasonographic measures disaggregated by sex

	Rest		0.75 min		1.25 min		1.75 min (iso‐time)		Peak	
	CO_2_	Exercise	CO_2_	Exercise	CO_2_	Exercise	CO_2_	Exercise	CO_2_	Exercise
Excursion (cm)										
M	1.57 (0.20)	1.74 (0.40)	2.89 (0.53)	1.96 (0.83)	3.56 (1.43)	2.36 (1.13)	3.98 (1.29)	2.63 (1.40)	4.93 (1.59)	5.40 (1.57)
F	2.21 (1.10)	1.85 (0.70)	2.53 (0.91)	2.23 (0.70)	2.72 (0.83)	2.32 (0.58)	3.15 (0.89)	2.30 (0.99)	3.94 (0.78)	4.70 (0.58)
Excursion time (s)										
M	1.53 (0.38)	1.40 (0.42)	1.64 (0.23)	1.34 (0.55)	1.74 (0.24)	1.49 (0.93)	1.60 (0.25)	1.73 (0.91)	1.56 (0.26)	1.31 (0.21
F	1.57 (0.49)	1.48 (0.35)	1.47 (0.43)	1.17 (0.23)	1.43 (0.41)	1.22 (0.28)	1.62 (0.32)	1.20 (0.14)	1.32 (0.20)	0.81 (0.08)
Excursion velocity (cm/s)										
M	1.07 (0.25)	1.26 (0.15)	1.79 (0.45)	1.48 (0.27)	2.07 (0.80)	1.75 (0.48)	2.55 (0.99)	1.52 (0.26)	3.23 (1.09)	4.10 (0.88)
F	1.35 (0.59)	1.22 (0.22)	1.71 (0.28)	1.91 (0.39)	1.89 (0.27)	1.91 (0.31)	2.05 (0.75)	1.87 (0.60)	2.99 (0.51)	5.76 (0.75)
Work (cmH_2_O × cm)										
M	5.4 (1.7)	8.9 (2.6)	12.8 (6.8)	12.5 (5.6)	14.4 (24.4)	21.8 (16.8)	32.4 (22.6)	22.1 (11.4)	43.3 (30.7)	54.4 (41.9)
F	12.3 (11.8)	10.9 (8.9)	14.3 (10.6)	17.9 (16.3)	16.8 (7.1)	16.1 (12.1)	24.8 (11.5)	17.9 (13.6)	38.6 (11.5)	76.3 (28.8)
Power (cmH_2_O × cm/s)										
M	3.6 (2.4)	6.5 (2.1)	8.1 (4.2)	9.6 (3.6)	14.0 (7.9)	15.9 (10.3)	21.0 (16.2)	13.1 (2.7)	32.9 (28.7)	69.3 (34.1)
F	7.1 (6.3)	6.7 (4.1)	9.3 (5.3)	14.8 (11.8)	11.3 (2.9)	12.0 (6.9)	16.5 (8.9)	14.0 (8.9)	29.1 (7.3)	91.8 (59.6)

Data are means (SD) for five males (M) and five females (F).

## 4 DISCUSSION

The aim of this study was to test whether the stabilising function of the human diaphragm would be altered differentially in response to involuntary augmented breaths induced with or without lower‐limb movements. We found that the diaphragm generated higher static (passive) pressure but moved significantly less (lower excursion) for equivalent levels of tidal volume and minute ventilation during lower‐limb cycle exercise compared with CO_2_‐rebreathe. Despite the apparent restriction to inspiration during exercise, diaphragm power output was relatively well preserved. Together, the findings support the notion that the power output of the diaphragm during stabilising tasks involving the lower‐limbs may be maintained via coordinated changes in contractile shortening.

### 4.1 Diaphragm excursion

It seems unlikely that the reduced excursion with lower‐body exercise was attributable to a change in the diaphragm position at end‐expiration because EE*P*_oe_ (an index of EELV) was maintained close to resting levels throughout both tasks. This finding is consistent with Henke et al. (1988), who showed that active expiration was not elicited, and hence EELV was unchanged from rest, at similar levels of minute ventilation for cycle exercise and CO_2_‐driven hyperpnoea when participants adopted a supine position. The most likely explanation for the lower excursion noted in the present study is a change in the diaphragm position at end‐inspiration. Indeed, EI*P*_oe_ (an index of EILV) tended to be less negative at equivalent time points throughout exercise compared with CO_2_‐rebreathe. The reason for the apparent decrease in diaphragm excursion is not entirely clear. One possibility is a change in the recruitment pattern of inspiratory muscles, although this seems unlikely because the relative contribution of ribcage muscles to inspiration (${\bar{P}}_{\mathrm{di}}$/${\bar{P}}_{\mathrm{oe}}$) was similar for both tasks at comparable levels of tidal breathing. Another possibility is an increase in the movement of the ultrasound transducer or a change in its orientation during exercise, although, again, we think this is unlikely for several reasons. First, angle‐independent (anatomic) M‐Mode sonography was used to assess the excursion of the diaphragm. With this approach, the cursor is positioned along the true axis of diaphragm excursion thereby leading to fewer orientation and translation errors (Orde et al., 2016). Second, the ultrasound transducer was anchored in relation to set anatomical landmarks to ensure the transducer was in a consistent orientation during scanning. Finally, we showed that the mean rates of rise (slopes) for the relationship between *V*_TI_ and diaphragm excursion (and *V*_TI_/*T*_I_ vs. excursion velocity) were similar for both tasks. We are confident therefore that the reduction in diaphragm excursion with exercise was not merely a result of measurement error. Potential mechanisms for the observed reduction in diaphragm excursion with exercise are discussed below.

### 4.2 Respiratory‐postural interactions

For equivalent levels of minute ventilation and tidal volume, the diaphragm generated higher force (${\bar{P}}_{\mathrm{di}}$) yet moved significantly less (lower excursion) during exercise compared with CO_2_‐rebreathe. By partitioning the active and passive components of ${\bar{P}}_{\mathrm{di}}$, it became evident that the higher ${\bar{P}}_{\mathrm{di}}$ during exercise was primarily due to an increase in the passive component (${\bar{P}}_{\mathrm{di},p}$). Whilst the active component (${\bar{P}}_{\mathrm{di},a}$) is determined by the tidal pressure swing, the passive component is determined by the amount of ‘bracing’ needed for trunk stabilisation and force generation. In other words, ${\bar{P}}_{\mathrm{di},p}$ is determined by the isometric (‘tonic’) activity of the diaphragm and other thoracic muscles (e.g., transverse abdominis) that passively elevate intra‐abdominal pressure. Because diaphragm excursion was lower during exercise compared with CO_2_‐rebreathe, it seems likely that the quasi‐isometric contractile pattern associated with increased postural demand constrained the range over which the diaphragm could move. In turn, the increased postural demand manifested as elevated intrathoracic and intra‐abdominal pressures (${\bar{P}}_{\mathrm{di}}$ and ${\bar{P}}_{\mathrm{ga}}$) and decreased diaphragm excursion. Interestingly, ${\bar{P}}_{\mathrm{di},p}$ returned to baseline levels within the first 30 s of exercise cessation whereas ${\bar{P}}_{\mathrm{di},a}$ remained elevated into recovery. Collectively, the findings suggest that the human diaphragm adopts a quasi‐isometric contractile pattern for the modulation of stabilising tasks that involve the lower‐limbs.

While our finding of a decrease in diaphragm excursion during dynamic lower‐limb exercise is novel, an effect of postural activities on diaphragm excursion has been reported previously. Kolar et al. (2012, 2010) demonstrated, using dynamic MRI, a greater increase in mean diaphragm excursion during brief, isometric flexion of upper or lower limbs against external resistance compared with tidal breathing alone, with the effect greatest for lower‐limb contractions. In addition, the authors noted that diaphragm excursion was most affected at the apex and crural regions of the diaphragm. Using a similar approach, Vostatek et al. (2013) showed that diaphragm excursion was increased when the lower limbs were loaded during isometric hip‐flexion in healthy control subjects. Again, the apex and crural regions of the diaphragm were the predominant contributors to the resultant diaphragm excursion. In all three studies, diaphragm excursion was assessed during a static postural task and compared with the response to tidal breathing at rest (Kolar et al., 2012, 2010; Vostatek et al., 2013). In contrast, we evaluated diaphragm excursion during a task with high external validity (i.e., lower‐limb cycle exercise) and compared the response to that induced by a task with low postural demand (i.e., CO_2_‐rebreathe). Moreover, we combined ultrasound‐derived measures of diaphragm shortening with measures of intrathoracic pressure to characterise more fully the stabilising function of the human diaphragm.

Several other studies have focused on respiratory–postural interactions. Although not directly comparable with the present study, the findings merit brief discussion. Gandevia and colleagues demonstrated that, in addition to its inspiratory role, the diaphragm contracts during postural tasks. The authors proposed that the neural input to the diaphragm during concurrent limb movement is the summation of ventilatory and postural inputs. That is, summation of ventilatory and postural inputs to the phrenic motoneurons causes the diaphragm to be ‘tonically’ active for postural support, with phasic modulation for diaphragm contraction during inspiration (Hodges & Gandevia, 2000a, 2000b). Later, the same group expanded their findings by imposing a postural task (single‐arm movement) during CO_2_‐induced hyperpnoea (Hodges et al., 2001). When ventilatory demand increased, the ‘tonic’ activity (EMG) of the diaphragm and the phasic activity with arm movement was reduced or absent. The authors suggested that diaphragm postural inputs to the phrenic motoneurons may be ‘gated’ or ‘occluded’ when descending respiratory drive is increased. The findings imply that the coordination between postural and ventilatory demands is compromised when the chemical drive to breathe is increased. While Hodges et al. (2001) provide convincing evidence of the mechanism for coordination of postural and respiratory functions of the trunk muscles, it is not entirely clear whether their findings extend to lower‐limb movements or, for that matter, any mode of dynamic exercise. Our study extends these previous findings by showing that the diaphragm contributes to trunk stability during dynamic lower‐limb exercise through increased ‘tonic’ activity with restricted movement.

### 4.3 Maintenance of contractile function

To further characterise the stabilising function of the diaphragm, we calculated power output during inspiration as the product of the mean change in active *P*_di_ (${\bar{P}}_{\mathrm{di},a}$), diaphragm excursion and 1/excursion time. Owing to concomitant reductions in inspiratory excursion and excursion time, excursion velocity (excursion/time) was similar for exercise and CO_2_‐rebreathe. In combination with time‐dependent increases in ${\bar{P}}_{\mathrm{di},a}$, the reductions in diaphragm excursion with exercise resulted in time‐dependent increases in diaphragm work (${\bar{P}}_{\mathrm{di},a}$ × excursion) and power output that were similar for the two tasks. Collectively, the results suggest that work and power of the diaphragm during stabilising tasks are maintained via coordinated changes in contractile shortening.

From rest to peak exercise, diaphragm power increased ∼12‐fold due to a ∼4‐fold increase in shortening velocity with only a ∼3‐fold increase in ${\bar{P}}_{\mathrm{di},a}$. These magnitudes of increase are similar to those reported by others. For instance, Aliverti et al. (1997), using opto‐electronic plethysmography (OEP) in combination with intrathoracic pressures, showed that diaphragm power increased up to 13‐fold from quiet breathing to 70% of maximum work‐rate during upright cycling. This increase was due to >6‐fold increases in shortening velocity with a less than doubling of *P*_di_. Further, we observed a ∼5‐fold increase in diaphragm power during CO_2_‐rebreathe that was due to a ∼3‐fold increase in shortening velocity with a less than doubling of *P*_di_. This increase in power during CO_2_‐rebreathe is similar to that reported by Romagnoli et al. (2004). Using OEP and intrathoracic pressures, those authors found a ∼5‐fold increase in diaphragm power at a level of CO_2_‐induced hypercapnia equivalent to that used in the present study ($P_{\mathrm{ETC}O_{2}}$ 55 mmHg). While the authors did not quantify the absolute changes in diaphragm shortening velocity, they did note a decline in the pressure‐to‐velocity ratio throughout the task, albeit at much higher levels of hypercapnia ($P_{\mathrm{ETC}O_{2}}$ 65−70 mmHg). Thus, our data support the notion that the diaphragm may function primarily as a flow generator, rather than as a pressure generator, during both dynamic lower‐limb exercise and CO_2_‐induced hypercapnia (i.e., hyperpnoea with and without postural demands). Other studies have assessed diaphragm power by combining measures of *P*_di_ and inspiratory duration with estimates of the volume displaced by excursion of the diaphragm made using lateral fluoroscopy (Finucane et al., 2005; Finucane & Singh, 2009). Using this approach, Finucane and Singh (2009) found a ∼5‐fold increase in diaphragm power during progressive hypercapnia in healthy individuals—an increase similar to that noted in the present study.

### 4.4 Technical considerations

Our estimates of diaphragm shortening are contingent upon the assumption that the motion of the right crural hemidiaphragm is representative of the average excursion for the entire muscle. Studies in dogs suggest that the two hemidiaphragms contract synergistically at rest and when contractile intensity (*P*_di_) is increased with phrenic nerve stimulation (De Troyer et al., 2003; see also De Troyer & Boriek, 2011). Whether the two hemidiaphragms contract synergistically during involuntary augmented breathing is not yet clear. We have also assumed that the crural diaphragm has a principal role in generating inspiratory pressure and airflow during augmented breathing. In general, the crural and costal segments are considered to have distinct roles. That is, the costal segment acts with appositional force on the lower ribcage for inspiratory expansion (De Troyer & Boriek, 2011), whereas the crural segment has a more important role in gastro‐oesophageal functions, such as retching and vomiting, and a significant role in providing a barrier during gastro‐oesophageal reflux (Menezes & Herbella, 2017; Miller, 1990). Analysis of intra‐breath profiles during progressive hypercapnia or hypoxia in dogs has shown that EMG activity of the crural segment precedes that of the costal segment during early inspiration and that the costal segment is fully shortened earlier during inspiration compared to the crural segment (Easton et al., 1995, 1993). When comparing the mean shortening of both costal and crural segments during the course of an entire hypercapnic rebreathe trial, however, there are no significant differences in fibre shortening, shortening velocity or EMG activity between the two segments (Fitting et al., 1985). We are confident, therefore, that our ultrasonographic measures of crural diaphragm shortening provide a good representation of the contractile function of the entire muscle during involuntary augmented breathing.

Several steps were taken to minimise measurement error and bias. First, all participants were screened thoroughly to mitigate the influence of pathology or central obesity. Second, all participants completed a thorough familiarisation trial to accustom them to the rebreathe and exercise protocols. Third, great effort was made to control the external environment by keeping the laboratory silent before, during and after each task, thereby preventing fluctuations in arousal level and the influence of participant–experimenter interaction (Homma & Masaoka, 2008; Spengler & Shea, 2001). Fourth, established guidelines were followed in the acquisition of ultrasound data (Laursen et al., 2021). Fifth, all analyses were conducted by the same investigator and were performed consistently in line with clearly defined standards (Orde et al., 2016). Finally, the study used a repeated‐measures design with a predetermined order of tasks (CO_2_‐rebreathe followed by exercise) to avoid the potential influence of prior respiratory muscle work or fatigue on subsequent responses to hypercapnia (Mador & Tobin, 1992). Whilst it is conceivable that hypercapnia may alter the ventilatory response to subsequent ventilatory challenge (Griffin et al., 2012), it seems unlikely that such a response would be induced with short‐term hypercapnia. Indeed, previous studies have shown that up to four repeated rebreathe tests over a 2 h period do not systematically alter the ventilatory response (Jensen et al., 2010; Sahn et al., 1977). A further consideration is that hypercapnic acidosis might elicit a reduction in diaphragm contractility, although, again, this seems unlikely on the basis that acute moderate hypercapnia has negligible effect on the *P*_di_ response to phrenic nerve stimulation (Mador et al., 1997; Vianna et al., 1993). In short, it appears doubtful that our results can be attributed to interaction or carry‐over effects.

Lastly, we did not consider biological sex to be relevant in the context of the present study and, therefore, did not design the study to detect sex‐based differences. Nevertheless, we did ensure equal representation of male and female participants and considered the ultrasonographic data sufficiently novel to disaggregate by sex (see Table 5). In general, the absolute values for diaphragm excursion at rest were larger for males compared with females, but similar to values reported previously (Boussuges et al., 2020). Moreover, the relative changes within each task were qualitatively similar for males and females. Thus, our findings are likely to extend to both sexes.

### 4.5 Conclusion

We have shown that the contractile function of the human diaphragm during dynamic lower‐limb exercise is relatively well preserved despite the possible restriction to inspiration that results from the increased requirement for postural stability. This supports the idea that the power output of the diaphragm during stabilising tasks involving the lower limbs is maintained via coordinated changes in contractile shortening. The findings further our understanding of the complex interplay between ventilatory and non‐ventilatory functions of the human diaphragm and may have significance in settings where the ventilatory and stabilising functions of the diaphragm must be balanced (e.g., rehabilitation).

## AUTHOR CONTRIBUTIONS

The experiments were performed at Brunel University London. Lee M. Romer and Camilla R. Illidi conceived and designed the study. Lee M. Romer and Camilla R. Illidi were involved in data collection and analysis. Lee M. Romer and Camilla R. Illidi drafted the article and critically revised for important intellectual content. Both authors approved the final version of the manuscript and agree to be accountable for all aspects of the work in ensuring that questions relating to the accuracy or integrity of any part of the work are appropriately investigated and resolved. All persons designated as authors qualify for authorship, and all those who qualify for authorship are listed.

## CONFLICT OF INTEREST

None.

## Supporting information

Statistical Summary Document

## Data Availability

The data that support the findings of this study are shown in the figures, tables and Supporting information.
